# Routine CYP2C19 Genotyping, Antiplatelet Prescription Changes, and Clinical Outcomes After PCI in Chinese Patients With Coronary Heart Disease: A Single‐Center Retrospective Study

**DOI:** 10.1155/crp/6267485

**Published:** 2026-09-28

**Authors:** Dan Niu, Mei Zhang, Hong Guan

**Affiliations:** ^1^ Department of Cardiology, Affiliated Hospital of Inner Mongolia Medical University, Hohhot, Inner Mongolia, China, nmgfy.com; ^2^ Department of Hematology, Ordos Central Hospital, Ordos, Inner Mongolia, China

**Keywords:** antiplatelet therapy, clopidogrel, coronary heart disease, CYP2C19, ticagrelor

## Abstract

**Background:**

CYP2C19 genotype can influence clopidogrel response, but the relationship between routine genotyping, real‐world P2Y12 inhibitor prescribing, and clinical outcomes remains uncertain.

**Aims:**

To characterize P2Y12 inhibitor prescription patterns before and after routine CYP2C19 genotyping and to describe ischemic and bleeding outcomes in a retrospective real‐world cohort.

**Methods:**

This single‐center retrospective study included 364 adults with coronary heart disease who underwent percutaneous coronary intervention (PCI) from January to December 2023. During the study period, CYP2C19 genotyping was routinely performed as part of the hospital’s antiplatelet management policy, and all 364 participants were genotyped. Prescription patterns before and after genotype results were compared; clinical outcome analyses were restricted to the 271 patients with complete linked postdischarge medication and outcome data.

**Results:**

Before genotyping, 273/364 patients (75.0%) received ticagrelor, and 91/364 (25.0%) received clopidogrel; after genotype results, 279/364 (76.6%) received clopidogrel, and 85/364 (23.4%) received ticagrelor. From the reported adjustment counts, 258 patients switched from ticagrelor to clopidogrel, 70 switched from clopidogrel to ticagrelor, and 36 remained on the same agent (exact McNemar *p* < 0.001). CYP2C19 genotypes were ∗1/∗1 in 132 (36.3%; normal metabolizer), ∗1/∗2 or ∗1/∗3 in 179 (49.2%; intermediate metabolizer), and two loss‐of‐function alleles in 53 (14.5%; poor metabolizer). In the 271‐patient outcome subset, event counts were low, and treatment strata were highly imbalanced. Fisher exact comparisons were therefore considered exploratory, and no causal effect of genotyping or drug selection was inferred.

**Conclusions:**

Routine CYP2C19 genotyping was associated with a substantial change in P2Y12 inhibitor prescribing in this single‐center cohort. Because treatment was physician‐directed and outcome data were sparse and incomplete for the full cohort, the clinical event comparisons should be interpreted as exploratory rather than evidence that genotype‐guided therapy reduced bleeding or ischemic events.

## 1. Introduction

Coronary heart disease (CHD), characterized by the narrowing or blockage of coronary arteries due to atherosclerosis, remains a significant global health challenge in the 21st century [1, 2]. As a leading cause of morbidity and mortality worldwide, approximately 17.5 million people died from cardiovascular diseases in 2019, with nearly half of these deaths attributed to CHD [2, 3]. Developing countries are experiencing rising CHD rates, driven by urbanization, sedentary lifestyles, and dietary changes [4, 5]. A national survey in China conducted in 2020 revealed that the prevalence of CHD among adults has reached 2.5%, translating to millions of affected individuals [6].

CHD is the underlying condition that can lead to stable angina (SA) as a chronic symptom. If the condition worsens, it can progress to acute coronary syndrome (ACS), which represents an acute event requiring immediate medical attention [7, 8]. The role of platelet activation and aggregation in atherothrombosis has been well‐established, making antiplatelet therapy a cornerstone in CHD management [9, 10]. Clinical evidence shows that long‐term use of antiplatelet drugs significantly reduces recurrent cardiovascular events and mortality in this high‐risk population [11, 12]. The effectiveness of antiplatelet therapy is primarily due to its ability to inhibit platelet activation and aggregation, thereby preventing thrombus formation on ruptured atherosclerotic plaques [13, 14]. Among antiplatelet agents, P2Y12 receptor antagonists are key in managing CHD, especially in ACS and percutaneous coronary interventions (PCIs) [15, 16]. Clopidogrel and ticagrelor are the two most widely prescribed P2Y12 receptor antagonists, each with unique pharmacological profiles [17–19].

Clopidogrel, a second‐generation thienopyridine, has been a mainstay of antiplatelet therapy for over two decades, supported by robust clinical evidence and a well‐established safety profile [20–22]. The CURE trial demonstrated a significant reduction in cardiovascular events in ACS patients treated with clopidogrel and aspirin [21]. However, its efficacy is limited by complex pharmacokinetics, requiring hepatic biotransformation to generate its active metabolite, influenced by genetic polymorphisms in the CYP2C19 gene [23, 24]. Approximately 15%–30% of patients exhibit inadequate platelet inhibition, a phenomenon known as “clopidogrel resistance” [23]. Ticagrelor, a more recent choice, offers several pharmacological advantages over clopidogrel, including more potent and consistent platelet inhibition with a faster onset and offset of action [25]. The PLATO trial showed that ticagrelor significantly reduces cardiovascular events and overall mortality compared to clopidogrel, leading to its recommendation as the preferred P2Y12 inhibitor in high‐risk ACS patients [25]. Despite the advantages, a higher incidence of noncoronary artery bypass grafting (CABG)–related bleeding and potential dyspnea in some patients were found in ticagrelor‐related clinical studies, while the twice‐daily dosing regimen may also impact its long‐term adherence compared to clopidogrel’s once‐daily dosing [26].

Choosing between clopidogrel and ticagrelor requires careful consideration of ischemic and bleeding risks, comorbidities, concomitant medications, and the expected effect of CYP2C19 genotype on clopidogrel activation [27, 28]. The 2022 CPIC guideline classifies CYP2C19 ∗1/∗1 as a normal‐metabolizer phenotype and recommends that intermediate and poor metabolizers undergoing ACS/PCI receive an alternative P2Y12 inhibitor such as ticagrelor or prasugrel when clinically appropriate [29]. Randomized trials including TAILOR‐PCI and POPular Genetics have further evaluated genotype‐guided selection strategies, although their designs and treatment algorithms differ from the present retrospective study [30, 31]. The contribution of routine genotyping to real‐world prescribing and outcomes therefore remains clinically relevant to describe without assuming causality.

This study aimed to describe antiplatelet prescribing before and after routine CYP2C19 genotyping in a single‐center real‐world PCI cohort, examine clinical factors associated with P2Y12 inhibitor selection, and explore documented ischemic and bleeding outcomes according to CYP2C19 phenotype and treatment.

## 2. Methods

### 2.1. Study Design and Population

The study retrospectively screened adult patients with CHD treated in the Department of Cardiology of the Affiliated Hospital of Inner Mongolia Medical University from January 2023 to December 2023. Eligible patients were (1) unrelated to each other; (2) aged 18 years or older; (3) clinically diagnosed with CHD, including ACS (unstable angina, STEMI, or NSTEMI) or SA; (4) treated with PCI; (5) not previously genotyped for CYP2C19 before the index episode; and (6) had complete baseline, medication, and CYP2C19 genotype data for the prescription analysis. Exclusion criteria were malignant tumor or acute/chronic infection; severe liver or kidney dysfunction, respiratory failure, or hematologic disease; use of anticoagulants, ticagrelor, or cilostazol within the two weeks before the index hospitalization; contraindication to aspirin or clopidogrel; or severe anemia, infection, or hyperthyroidism. During the study period, CYP2C19 genotyping was routinely performed as part of the center’s antiplatelet management policy for PCI patients; all 364 participants included in this analysis underwent genotyping. The study was approved by the Ethics Committee of the Affiliated Hospital of Inner Mongolia Medical University (approval no. [to be confirmed before resubmission]).

### 2.2. Data Collection

Medical records of eligible patients were retrieved and reviewed. Demographics, clinical characteristics, medication history, CYP2C19 genotype results, postgenotyping P2Y12 inhibitor prescriptions, and documented ischemic and bleeding events were abstracted into an electronic dataset by two investigators. Tables 1 and 2 compare characteristics between clopidogrel‐ and ticagrelor‐treated patients at the pregenotyping and postgenotyping time points, respectively; they are not intended as a before‐versus‐after comparison of patient demographics.

**TABLE 1:** Characteristics according to P2Y12 inhibitor before CYP2C19 genotype availability.

	Clopidogrel (*N* = 91)	Ticagrelor (*N* = 273)	*p* value
Age (years, mean ± SD)	60.69 ± 8.65	60.62 ± 9.08	0.909
Male, *n* (%)	60 (65.9%)	191 (70.0%)	0.472
Ethnic minority, *n* (%)	26 (28.6%)	57 (20.9%)	0.130
Hypertension	66 (72.5%)	195 (71.4%)	0.840
Diabetes	11 (12.1%)	46 (16.8%)	0.279
Hyperlipidemia	10 (11.0%)	34 (12.5%)	0.710
Atrial fibrillation	1 (1.1%)	17 (6.2%)	0.053
Peripheral vascular disease	1 (1.1%)	9 (3.3%)	0.462
Smoking	33 (36.3%)	113 (41.4%)	0.387
Alcohol use	25 (27.5%)	94 (34.4%)	0.220
Bleeding risk factor (s)	19 (20.9%)	53 (19.4%)	0.761
TIA or stroke	2 (2.2%)	7 (2.6%)	> 0.999
End‐stage renal disease	3 (3.3%)	4 (1.5%)	0.373
Age ≥ 75 years	7 (7.7%)	16 (5.9%)	0.534
Weight ≤ 60 kg	6 (6.6%)	23 (8.4%)	0.576
History of major bleeding event	1 (1.1%)	3 (1.1%)	> 0.999
Concomitant discharge medication			
β‐Blocker	77 (84.6%)	226 (82.8%)	0.685
Statin	91 (100%)	273 (100%)	> 0.999
Proton pump inhibitor	85 (93.4%)	253 (92.7%)	0.814
ACEI or ARB	81 (89.0%)	242 (88.6%)	0.924
Anticoagulant	14 (15.4%)	54 (19.8%)	0.351

Abbreviations: ACEI, angiotensin‐converting enzyme inhibitor; ARB, Angiotensin II receptor blocker; TIA, transient ischemic attack.

**TABLE 2:** Characteristics according to P2Y12 inhibitor after CYP2C19 genotype availability.

	Clopidogrel (*N* = 279)	Ticagrelor (*N* = 85)	*p* value
Age (years, mean ± SD)	60.77 ± 9.03	60.82 ± 8.89	0.965
Male, *n* (%)	198 (71.0%)	53 (62.4%)	0.133
Ethnic minority, *n* (%)	57 (20.4%)	26 (30.6%)	0.051
Hypertension	198 (71%)	63 (74.1%)	0.572
Diabetes	45 (16.1%)	12 (14.1%)	0.655
Hyperlipidemia	37 (13.3%)	7 (8.2%)	0.213
Atrial fibrillation	17 (6.1%)	1 (1.2%)	0.085
Peripheral vascular disease	6 (2.2%)	4 (4.7%)	0.252
Smoking	117 (41.9%)	29 (34.1%)	0.209
Alcohol use	93 (33.3%)	26 (30.6%)	0.693
Bleeding risk factor (s)	54 (19.4%)	18 (21.2%)	0.756
TIA or stroke	9 (3.2%)	0 (0%)	0.124
End‐stage renal disease	4 (1.4%)	3 (3.5%)	0.362
Age ≥ 75 years	17 (6.1%)	6 (7.1%)	0.749
Weight ≤ 60 kg	21 (7.5%)	8 (9.4%)	0.574
History of major bleeding event	3 (1.1%)	1 (1.2%)	> 0.999
Concomitant discharge medication			
β‐Blocker	231 (82.8%)	72 (84.7%)	0.680
Statin	279 (100%)	85 (100%)	> 0.999
Proton pump inhibitor	259 (92.8%)	79 (92.9%)	0.973
ACEI or ARB	249 (89.2%)	74 (87.1%)	0.576
Anticoagulant	55 (19.7%)	13 (15.3%)	0.360
CYP2C19 phenotype			< 0.001
Normal/intermediate metabolizer	276 (98.9%)	35 (41.2%)	
Poor metabolizer	3 (1.1%)	50 (58.8%)	

Abbreviations: ACEI, angiotensin‐converting enzyme inhibitor; ARB, Angiotensin II receptor blocker; TIA, transient ischemic attack.

### 2.3. Genotyping, Treatment Timing, and Outcome Ascertainment

Genotyping was performed during the index hospitalization. The exact turnaround time from PCI/genotyping to genotype‐result availability was not consistently recorded in the source dataset and therefore could not be standardized or analyzed. For this retrospective analysis, the pregenotyping P2Y12 inhibitor was the agent documented before genotype‐result availability, and the postgenotyping P2Y12 inhibitor was the agent documented after the result became available and before discharge. The exact interval between result availability and treatment switching was not consistently retrievable. Ischemic and bleeding events were abstracted from available follow‐up records. Because ischemic event subtype and Bleeding Academic Research Consortium (BARC) grade were not consistently available in the source dataset, ischemic events are reported as clinician‐documented ischemic events and bleeding events as clinician‐documented bleeding events without subtype or BARC stratification. Individual follow‐up duration was not uniformly recoverable; consequently, outcome analyses were restricted to patients with complete linked postdischarge medication and outcome data and were treated as exploratory.

### 2.4. Statistical Analysis

Descriptive statistics were used to summarize demographic and clinical characteristics. Frequencies and percentages were calculated for categorical variables, and means with standard deviations (SDs) were used for continuous variables. Chi‐square tests or Fisher’s exact tests were used for categorical comparisons, and independent‐samples *t* tests for continuous variables. The paired change in P2Y12 inhibitor from before to after genotype availability was evaluated with an exact McNemar test using the transition counts that could be reconstructed from the reported adjustment data. Because the clinical outcome strata contained very small cells and zero‐event groups, outcome comparisons were limited to two‐sided Fisher exact tests and were considered exploratory. A multivariable logistic model for bleeding was not retained because sparse/zero‐event cells would yield unstable, separation‐prone estimates. Statistical significance was defined as a two‐sided *p* < 0.05. Analyses were originally performed in SPSS Version 24.0 (IBM, Armonk, NY, USA); exact contingency‐table calculations in the revision were rechecked from the reported tabulated counts.

## 3. Results

### 3.1. Baseline Characteristics and Initial Drug Selection Before CYP2C19 Genotyping

A total of 364 patients were included, with a mean age of 60.79 ± 8.98 years. Among them, 251 (69.0%) were male, and 83 (22.8%) were from ethnic minority groups, predominantly Mongolian (79.5%). The most common comorbidities included hypertension in 261 patients (71.7%), diabetes in 57 (15.7%), and dyslipidemia in 44 (12.1%). A total of 146 patients (40.1%) were smokers, 119 (32.7%) reported alcohol use, and 72 (19.8%) had recorded bleeding risk factors. Before genotyping, 273 patients (75.0%) received ticagrelor, and 91 (25.0%) received clopidogrel. No measured baseline characteristic showed a statistically significant association with the pregenotyping P2Y12 inhibitor choice (Table 1).

### 3.2. Clinical Characteristics and Drug Selection After CYP2C19 Genotyping

After genotype results became available, 279 patients (76.6%) were prescribed clopidogrel and 85 (23.4%) ticagrelor. The paired prescription transition reconstructed from the reported adjustment counts was as follows: ticagrelor to clopidogrel, *n* = 258; clopidogrel to ticagrelor, *n* = 70; clopidogrel unchanged, *n* = 21; and ticagrelor unchanged, *n* = 15. This represented a significant shift in the paired prescription distribution (exact McNemar *p* < 0.001). Numerically, 17/18 patients with atrial fibrillation and all 9 patients with a recorded history of TIA or stroke received clopidogrel after genotyping, although neither clinical characteristic was significantly associated with postgenotyping drug selection (*p* = 0.085 and *p* = 0.124, respectively). CYP2C19 phenotype was strongly associated with postgenotyping treatment (*p* < 0.001) (Table 2).

### 3.3. Diagnosis and Antiplatelet Agent Selection

Antiplatelet selection did not differ significantly between patients with ACS and those with SA at either time point. Before genotyping, clopidogrel was used in 75/305 ACS patients (24.6%) and 16/59 SA patients (27.1%); after genotyping, clopidogrel was used in 237/305 ACS patients (77.7%) and 42/59 SA patients (71.2%) (both *p* > 0.05) (Table 3).

**TABLE 3:** Clinical presentation and P2Y12 inhibitor selection/adjustment.

	ACS	SA	*p* value
P2Y12 inhibitor before CYP2C19 genotype availability			
Clopidogrel	75 (24.6%)	16 (27.1%)	0.681
Ticagrelor	230 (75.4%)	43 (72.9%)	
P2Y12 inhibitor after CYP2C19 genotype availability			
Clopidogrel	237 (77.7%)	42 (71.2%)	0.279
Ticagrelor	68 (22.3%)	17 (28.8%)	
Normal/intermediate metabolizer	263	48	
Antiplatelet agent adjustment	231 (87.8%)	45 (93.8%)	0.233
Step‐down	214 (92.6%)	41 (91.1%)	0.758
Step‐up	17 (7.4%)	4 (8.9%)	
Poor metabolizer	42	11	
Antiplatelet agent adjustment	41 (97.6%)	11 (100%)	> 0.999
Step‐down	3 (7.3%)	0 (0%)	> 0.999
Step‐up	38 (92.7%)	11 (100%)	

### 3.4. Genotypes of CYP2C19

CYP2C19 genotyping identified 132 patients (36.3%) with ∗1/∗1, classified as normal metabolizers according to CPIC nomenclature; 179 (49.2%) with ∗1/∗2 or ∗1/∗3, classified as intermediate metabolizers; and 53 (14.5%) with ∗2/∗2, ∗2/∗3, or ∗3/∗3, classified as poor metabolizers (Table 4) [29].

**TABLE 4:** CYP2C19 genotype distribution and phenotype classification.

CYP2C19 genotype	Phenotype	*n*	Proportion (%)
∗1/∗1	Normal metabolizer	132	36.3
∗1/∗2, ∗1/∗3	Intermediate metabolizer	179	49.2
∗2/∗2, ∗2/∗3, ∗3/∗3	Poor metabolizer	53	14.5

### 3.5. Drug Adjustment Based on Genotype and Clinical Characteristics

In the source prescription‐adjustment analysis, normal and intermediate metabolizers were combined (*n* = 311). Among them, 276 (88.7%) had a change in P2Y12 inhibitor: 255 (92.4% of those who changed) switched from ticagrelor to clopidogrel and 21 (7.6%) from clopidogrel to ticagrelor. Among 53 poor metabolizers, 52 (98.1%) had a treatment change: 49 (94.2% of those who changed) switched to ticagrelor and 3 (5.8%) to clopidogrel. Adjustment rates and directions did not significantly differ between ACS and SA groups (Table 3). The combination of normal and intermediate phenotypes in this prescription table reflects the structure of the source summary data and should not be interpreted as biological equivalence between these phenotypes.

### 3.6. Clinical Outcomes in the Documented Follow‐Up Subset

Complete linked postdischarge medication and outcome records were available for 271 of 364 participants (74.5%): 102/132 normal metabolizers, 129/179 intermediate metabolizers, and 40/53 poor metabolizers. Thus, 93 participants (30 normal, 50 intermediate, and 13 poor metabolizers) were retained in the prescription analyses but were not represented in Table 5 because complete linked outcome data were not available in the source outcome dataset. Among normal metabolizers, composite ischemic/bleeding events occurred in 5/99 (5.1%) patients receiving clopidogrel and 2/3 (66.7%) receiving ticagrelor (two‐sided Fisher exact *p* = 0.012); bleeding alone occurred in 2/99 versus 1/3 (*p* = 0.086). Among intermediate metabolizers, composite events occurred in 2/101 (2.0%) receiving clopidogrel and 5/28 (17.9%) receiving ticagrelor (*p* = 0.005); bleeding alone occurred in 1/101 versus 3/28 (*p* = 0.032). No ischemic or bleeding events were recorded among the 3 poor metabolizers receiving clopidogrel or the 37 receiving ticagrelor. Because treatment was physician‐directed and several treatment strata were extremely small, these unadjusted comparisons are exploratory and should not be interpreted as causal treatment effects (Table 5).

**TABLE 5:** Exploratory clinical outcomes in patients with complete linked outcome data (*n* = 271).

CYP2C19 phenotype and antiplatelet agent	*n*	Ischemic event, *n* (%)	Bleeding event, *n* (%)	Composite event, *n* (%)	Fisher′s exact *p* ^∗^
Normal metabolizer + clopidogrel	99	3 (3.0%)	2 (2.0%)	5 (5.1%)	0.012
Normal metabolizer + ticagrelor	3	1 (33.3%)	1 (33.3%)	2 (66.7%)	

Intermediate metabolizer + clopidogrel	101	1 (1.0%)	1 (1.0%)	2 (2.0%)	0.005
Intermediate metabolizer + ticagrelor	28	2 (7.1%)	3 (10.7%)	5 (17.9%)	

Poor metabolizer + clopidogrel	3	0	0	0	Not estimable
Poor metabolizer + ticagrelor	37	0	0	0	

*Note:* Bleeding‐only Fisher’s exact *p* values were 0.086 for normal metabolizers and 0.032 for intermediate metabolizers. Outcome analysis includes 271/364 participants with complete linked postdischarge medication and outcome data; sparse and imbalanced strata make these comparisons exploratory.

^∗^ *p* values are two‐sided Fisher exact tests comparing clopidogrel versus ticagrelor within the phenotype for the composite ischemic/bleeding endpoint.

## 4. Discussion

This retrospective real‐world study found a marked change in P2Y12 inhibitor prescribing after routine CYP2C19 genotype results became available. The principal contribution of the study is descriptive: it documents how prescribing changed in routine practice and how clinical events were distributed in the subset with linked outcome data. The observational design, physician‐directed treatment, incomplete outcome data for the full cohort, and highly imbalanced genotype‐treatment strata preclude causal statements that genotyping itself reduced bleeding or ischemic events.

The revised interpretation also distinguishes CYP2C19 phenotypes according to current CPIC terminology. CYP2C19 ∗1/∗1 is a normal‐metabolizer phenotype, whereas ∗1/∗2 and ∗1/∗3 are intermediate‐metabolizer phenotypes associated with reduced clopidogrel activation [29]. For patients with ACS and/or PCI, CPIC recommends standard‐dose clopidogrel when it is otherwise appropriate in normal, rapid, and ultrarapid metabolizers, but recommends an alternative P2Y12 inhibitor in intermediate and poor metabolizers when clinically appropriate [29]. This distinction is important because our source prescription‐adjustment table combined normal and intermediate metabolizers, and that grouping reflects the available summary data rather than a claim that the two phenotypes have equivalent clopidogrel response. In POPular Genetics, a genotype‐guided de‐escalation strategy using clopidogrel in patients without CYP2C19 loss‐of‐function alleles and ticagrelor/prasugrel in loss‐of‐function carriers was noninferior for thrombotic outcomes and reduced bleeding compared to routine potent P2Y12 inhibition [31]. In TAILOR‐PCI, genotype‐guided escalation among loss‐of‐function carriers did not meet the prespecified primary superiority threshold, although the direction of effect favored genotype‐guided therapy [30]. These trials support the clinical relevance of CYP2C19 genotype but do not validate a conclusion that clopidogrel is superior for intermediate metabolizers in the present observational cohort.

The lower observed bleeding counts in some clopidogrel‐treated strata should also be interpreted in light of pharmacologic potency and confounding by indication. Clopidogrel generally produces less potent platelet inhibition than ticagrelor, which can contribute to lower bleeding rates independent of genotype. In our normal‐metabolizer outcome subset, the bleeding comparison (2/99 with clopidogrel vs. 1/3 with ticagrelor) was not statistically significant by Fisher exact testing (*p* = 0.086), and the ticagrelor subgroup contained only three patients. Although the intermediate‐metabolizer bleeding comparison was nominally significant (1/101 vs. 3/28; *p* = 0.032), treatment was not randomized, and current CPIC guidance favors an alternative P2Y12 inhibitor rather than clopidogrel for intermediate metabolizers undergoing ACS/PCI [29]. These findings therefore generate hypotheses but should not be used to infer treatment superiority.

Several limitations should be acknowledged. First, this was a retrospective, single‐center observational study without a contemporaneous nongenotyped control group; treatment was physician‐directed, creating substantial potential for confounding by indication and preventing causal attribution to genotyping. Second, exact genotype turnaround time, the interval from result availability to treatment switching, and individual follow‐up duration were not consistently recoverable from the source dataset. Third, complete linked outcome data were available for only 271/364 participants, and the reason for missing outcome linkage could not be further reconstructed from the available summary data; selection bias in the outcome subset is therefore possible. Fourth, ischemic event subtypes and BARC bleeding grades were not consistently available, preventing event‐specific or severity‐specific analyses. Fifth, outcome counts were low, and several genotype‐treatment strata were very small or had zero events, making multivariable regression unstable and limiting statistical power. Finally, unmeasured factors including adherence, socioeconomic factors, physician preference, and other clinical risk features may have influenced both treatment choice and outcomes.

Future work should use prospective multicenter designs with standardized genotype turnaround times, prespecified treatment algorithms, uniform follow‐up, adjudicated ischemic endpoints, BARC‐classified bleeding, and sufficient sample size within each CYP2C19 phenotype. Such studies could more reliably assess clinical effectiveness and cost‐effectiveness of routine genotype‐informed antiplatelet selection.

## 5. Conclusion

In conclusion, routine CYP2C19 genotyping was associated with a substantial change in P2Y12 inhibitor prescribing after PCI in this single‐center cohort. The study provides a real‐world description of genotype‐associated prescribing patterns, but the available clinical outcome data are exploratory because of nonrandom treatment selection, incomplete follow‐up linkage, sparse events, and imbalanced subgroups. These findings support further prospective evaluation of standardized genotype‐informed strategies rather than a causal conclusion that genotyping or clopidogrel reduced bleeding risk.

**Nomenclature**
ACS: Acute coronary syndrome
CABG: Coronary artery bypass grafting
CPIC: Clinical Pharmacogenetics Implementation Consortium
CHD: Coronary heart disease
LOF: Loss‐of‐function
NSTEMI: Non–ST‐segment elevation myocardial infarction
PCI: Percutaneous coronary intervention
SA: Stable angina
STEMI: ST‐segment elevation myocardial infarction
TIA: Transient ischemic attack

## Funding

This work was funded by the research project of Inner Mongolia Medical University Affiliated Hospital (Grant no. 2023NYFYTS004).

## Conflicts of Interest

The authors declare no conflicts of interest.

## Data Availability

The data that support the findings of this study are available from the corresponding author upon reasonable request.
